# Southern Light

**Published:** 1878-05

**Authors:** 


Elsewhere is mention made of a pamphlet by Prof. Donaldson, and in that notice we took occasion to invite correspondence on the subject of *Southern Light*, and its influence on the health of the dentist. We will be glad to hear from all who have moved their offices to the *south side* of the house, and who feel that the change has helped them. The health of the dentist is an exceedingly important subject, and if there is advantage in the position let us find out all about it.

